# A Novel Localization of METTL7A in Bergmann Glial Cells in Human Cerebellum

**DOI:** 10.3390/ijms24098405

**Published:** 2023-05-07

**Authors:** América Vera-Montecinos, Jordi Galiano-Landeira, Mònica Roldán, Francisco Vidal-Domènech, Enrique Claro, Belén Ramos

**Affiliations:** 1 Psiquiatria Molecular, Parc Sanitari Sant Joan de Déu, Institut de Recerca Sant Joan de Déu, Dr. Antoni Pujadas, 42, 08830 Sant Boi de Llobregat, Spain; amevera@gmail.com (A.V.-M.); jordi.galiano@sjd.es (J.G.-L.); francisco.vidal@sjd.es (F.V.-D.); 2 Unitat de Microscòpia Confocal i Imatge Cel·lular, Servei de Medicina Genètica i Molecular, Institut Pediàtric de Malalties Rares (IPER), Hospital Sant Joan de Déu, Esplugues de Llobregat, 08950 Barcelona, Spain; monica.roldan@sjd.es; 3 Institut de Recerca Sant Joan de Déu, Esplugues de Llobregat, 08950 Barcelona, Spain; 4 Departament de Bioquímica i Biologia Molecular, Facultat de Medicina, Universitat Autònoma de Barcelona, 08193 Bellaterra, Spain; 5 Institut de Neurociències, Universitat Autònoma de Barcelona, 08193 Bellaterra, Spain; 6 Centro de Investigación Biomédica en Red de Salud Mental, CIBERSAM (Biomedical Network Research Center of Mental Health), Institute of Health Carlos III, 28029 Madrid, Spain; 7 Faculty of Medicine, University of Vic-Central University of Catalonia, 08500 Vic, Spain

**Keywords:** METTL7A, schizophrenia, postmortem cerebellum, Bergmann glia, glia limitans, cerebrospinal fluid-brain barrier

## Abstract

Methyltransferase-like protein 7A (METTL7A) is a member of the METTL family of methyltransferases.Little information is available regarding the cellular expression of METTL7A in the brain. METTL7A is commonly located in the endoplasmic reticulum and to a lesser extent, in the lipid droplets of some cells. Several studies have reported altered protein and RNA levels in different brain areas in schizophrenia. One of these studies found reduced protein levels of METTL7A in the cerebellar cortex in schizophrenia and stress murine models. Since there is limited information in the literature about METTL7A, we characterized its cellular and subcellular localizations in the human cerebellum using immunohistochemical analysis with laser confocal microscopy. Our study reveals a novel METTL7A localization in GFAP-positive cells, with higher expression in the end-feet of the Bergmann glia, which participate in the cerebrospinal fluid–brain parenchyma barrier. Further 3D reconstruction image analysis showed that METTL7A was expressed in the contacts between the Bergmann glia and Purkinje neurons. METTL7A was also detected in lipid droplets in some cells in the white matter. The localization of METTL7A in the human cerebellar glia limitans could suggest a putative role in maintaining the cerebellar parenchyma homeostasis and in the regulation of internal cerebellar circuits by modulating the synaptic activity of Purkinje neurons.

## 1. Introduction

Methyltransferase-like protein 7A (METTL7A) is a member of the METTL family of methyltransferases. METTL7A has an m^6^A methyltransferase activity to RNA [1]. In addition, the overexpression of METTL7A induced an increase in DNA methylation [2]. Recently, some authors have also suggested that METTL7A has S-thiol methyltransferase activity, with a possible impact on drug metabolism [3]. In addition, it has also been shown to interact with proteins with different enzymatic activities (e.g., histone- or actin-modifying proteins), DNA/RNA-binding proteins, and signaling proteins, among others [4]. METTL7A has been reported to localize mainly in the endoplasmic reticulum [5,6,7] and to a lesser extent, in lipid droplets [5,8,9]. METTL7A has been shown to participate in osteogenic differentiation and cell survival [2,10], lipid droplet formation [5,6,9,11], and immune processes [7,12,13]. Indeed, Gene Ontology classifies the biological function of METTL7A in neutrophil degranulation and its subcellular localization in lipid droplets. Studies in different areas of the human brain have revealed changes in METTL7A in schizophrenia [14,15,16]. One of these studies identified a reduction in METTL7A protein levels in the cerebellar cortex in schizophrenia subjects and stress mouse models, respectively [14]. Distinct hypotheses have been postulated regarding the origin of schizophrenia [17,18]; among these models, the idea of immune dysregulation has recently been gaining importance [18,19,20,21]. Since METTL7A plays a role in the immune response, METTL7A could contribute to the central immune activation described in schizophrenia [7,12,13,18,19]. Nevertheless, METTL7A function and cell expression in the brain remain unexplored.

The altered immune response in the central nervous system could lead to the release of proinflammatory molecules and, among other effects, cause a permeabilization of the blood–brain barrier (BBB) [22]. In the context of cerebral barriers, the cerebellum is separated from the cerebrospinal fluid (CSF) by a barrier formed by the pia mater and the end-feet of Bergmann glial cells, which constitute the glia limitans [23]. The pia mater is the inner meningeal layer, constituted by leptomeningeal cells that cover the outer brain and the cerebellum [24]. Thus, the glia limitans form an intimate contact between the cerebellar parenchyma and the pia mater [25]. Thus, an altered immune response could impact the molecular integrity of the cerebellar barrier and contribute to parenchyma damage where the Purkinje neurons and Bergmann glial cells are located. Despite the possible role of METTL7A in the immune response in the cerebellum, the cellular expression and localization of METTL7A in the cerebellar cortex remain unknown. Our study aims to characterize the localization of METTL7A in human cerebellar tissue using laser confocal microscopy.

## 2. Results

### 2.1. Characterization of METTL7A in Human Cerebellar Tissue 

We first confirmed the proper tissue preservation of the human cerebellar sample using hematoxylin-eosin staining (Figure S1A). We also optimized the blocking conditions to avoid background signals from secondary antibodies (Figure S1B).

**Figure 1:**
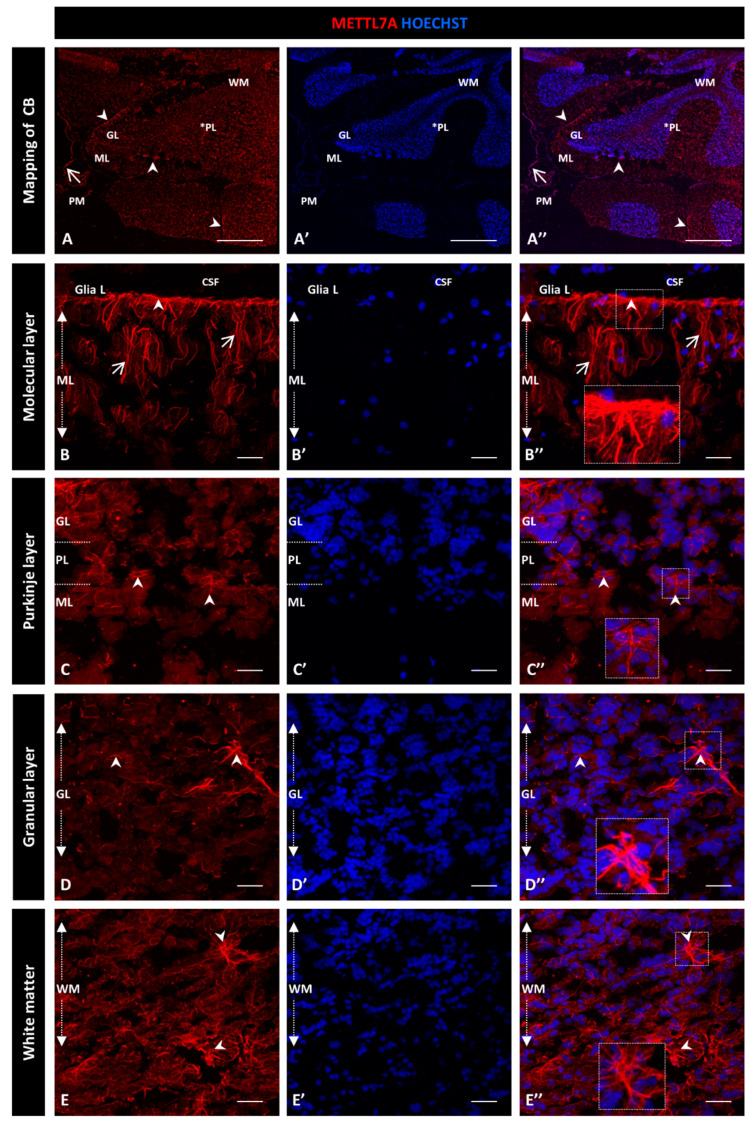
Immunohistochemistry of METTL7A in human cerebellum using a polyclonal antibody: (**A**–**E**) Images show the immunodetection of METTL7A (red). (**A**–**A″**) Mapping of cerebellum, showing higher METTL7A labeling in the apical region (arrowheads). Asterisk indicates PL. (**B**–**B″**) Strong immunoreactivity of METTL7A in the molecular layer (arrows) and the apical region (arrowheads). (**C**–**C″**) Image of the Purkinje cell layer, possibly showing METTL7A in the Bergmann glial cells (arrowheads). (**D**–**D″**) The granular layer shows low METTL7A immunoreactivity but is high in some cells (arrowheads). (**E**–**E″**) White matter shows high METTL7A immunoreactivity. (**A′**,**B′**,**C′**,**D′**,**E′**) Nuclei were stained with Hoechst 33342 (blue). (**A**–**E**) Tissue thickness—7 µm. (**A**) Scale bar—1 mm and 10× magnification. (**B**–**E**) Scale bar—4 µm, 20× magnification. Insets are zoomed in twofold. **CB**: cerebellum; **CSF**: cerebrospinal fluid; **GL**: granular layer; **ML**: molecular layer; **PM**: pia mater; **PL**: Purkinje cell layer; **WM**: white matter.

For this qualitative study, we used two different METTL7A antibodies and obtained similar expression patterns: a polyclonal antibody (Figure 1) and a monoclonal antibody (Figure S2). Figure 1A–A″ show a general view of a sagittal section of the cerebellum. From the innermost to the outermost layers are the white matter, the granular layer, and the Purkinje layer, which is between the granular and molecular layers and is indicated with an asterisk. Figure 1A″ shows the pia mater covering each cerebellar lobule. We found that METTL7A labeling was strong in the apical region of the molecular layer, where it showed a fibrillary profile across the molecular layer (Figure 1B–B″ for the polyclonal and Figure S2A–A″ for the monoclonal antibody). We observed moderate immunodetection for METTL7A in the Purkinje layer (Figure 1A,C–C″ and Figure S2B–B″), low in the granular layer (Figure 1A,D–D” and Figure S2C–C″), and high in white matter (Figure 1A,E–E″ and Figure S2D–D″).

In addition, we explored the pattern expression of METTL7A in the cerebellum in open online resources. We ran an analysis in the Human Cell Landscape and DropViz software [26,27]. We first performed a query in the Human Cell Landscape software in order to detect the METTL7A transcript in the human cerebellum. According to the human source, METTL7A is expressed in 40% of Bergmann glial cells (*p*-value = 1.5582 × 10^−155^) and 38% of astrocytes (*p*-value = 1). In mouse cerebellum using DropViz, we obtained the top 10 cell type clusters that express *mettl7a1* in the cerebellum. Significantly expressed cell type clusters are shown in Supplementary Data S1. *mettl7a1* is expressed the highest in the choroid plexus and oligodendrocyte/polydendrocyte clusters. The third cell cluster with highest *mettl7a1* expression comprises the Bergmann glia, which have the lowest *p*-value reported. The rest of the cell clusters are granular neurons, endothelial cells, interneurons, and Purkinje neurons.

### 2.2. Co-Immunostaining of METTL7A and GFAP in Human Cerebellar Tissue

**Figure 2:**
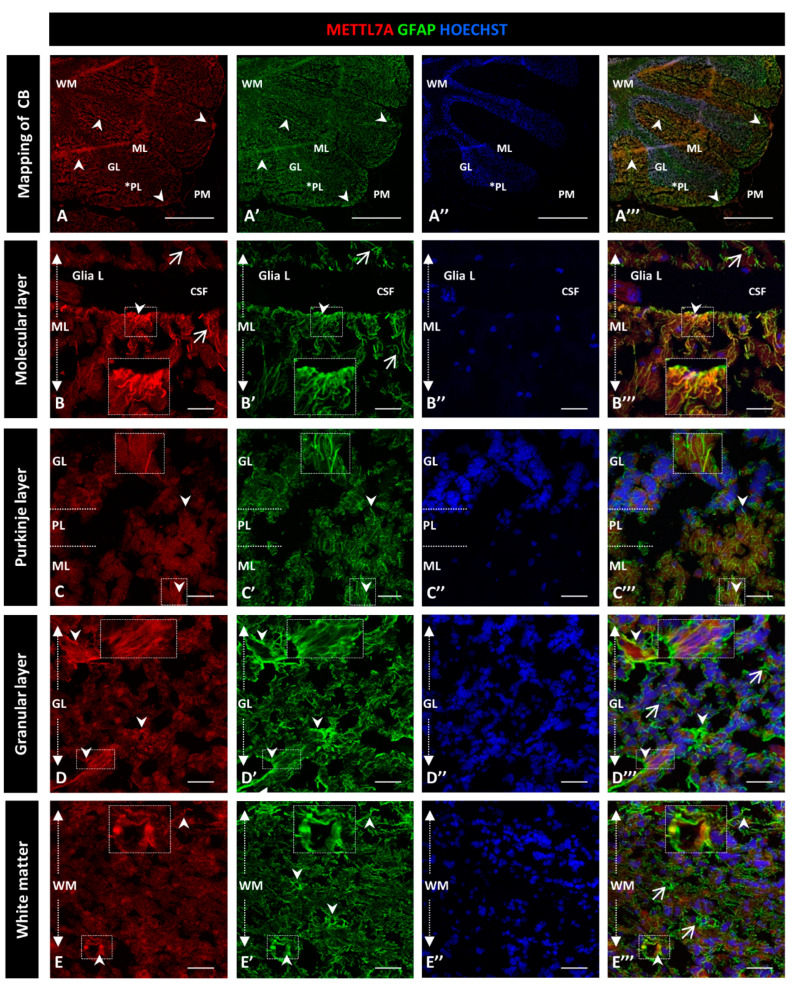
Immunodetection of METTL7A and GFAP in the human cerebellum. (**A**–**E**) Images show the immunodetection of METTL7A using polyclonal antibody (red) and GFAP using monoclonal antibody (green). (**A**–**A‴**) Mapping of the human cerebellum. (**A**) Immunodetection of METTL7A (arrowheads). Asterisk indicates PL. (**A′**) Immunoreactivity for GFAP (arrowheads). (**A‴**) Image shows the colocalization of both proteins (arrowheads). (**B**) Immunoreactivity of METTL7A distributed across the molecular layer (arrows) and strong immunoreactivity in the apical region (arrowheads). (**B′**) High immunodetection of GFAP in the Bergmann glia (arrows) and the apical region (arrowheads). (**B‴**) Colocalization of METTL7A and GFAP in the Bergmann glia (arrow) and strong immunodetection in the apical region (arrowheads). (**C**) Immunoreactivity of METTL7A in the Purkinje layer, possibly in the Bergmann glia (arrowheads). (**C′**) Note positive cells for GFAP in the Purkinje layer (arrowheads). (**C‴**) Colocalization of METTL7A and GFAP in the Purkinje layer, possibly in the cytoplasm of Bergmann glia (arrowheads). (**D**) Low immunoreactivity in the granular layer (arrowheads). (**D′**) Strong immunodetection of GFAP in cells of the granular layer. (**D‴**) Merge of METTL7A and GFAP in the granular layer; arrowheads indicate colocalization of METTL7A and GFAP and arrows show positive immunodetection for GFAP. (**E**) Panel shows the expression of METTL7A (arrowheads) in white matter. (**E′**) Immunodetection for GFAP (arrowheads) in the white matter. (**E‴**) Merge of METTL7A and GFAP in white matter and their colocalization (arrowheads) and positive expression of GFAP (arrows). (**A″**,**B″**,**C″**,**D″**,**E″**) Nuclei stained with Hoechst 33342 (blue). (**A**–**D**) Tissue thickness—7 µm. (**A**) Scale bar—1 mm, 10× magnification. (**B**–**E**) Scale bar—4 µm, 20× magnification. Insets are zoomed in twofold. **CB**: cerebellum; **CSF**: cerebrospinal fluid; **GL**: granular layer; **Glia L**: glia limitans; **ML**: molecular layer; **PL**: Purkinje layer; **PM**: pia mater; **WM**: white matter.

**Figure 3:**
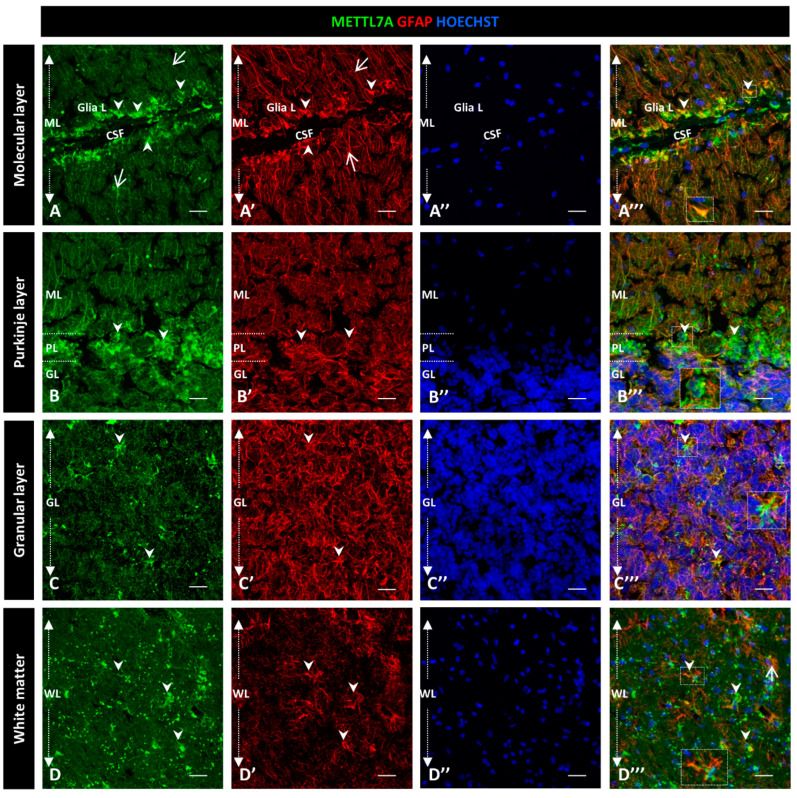
Immunodetection of METTL7A and GFAP in human cerebellum. (**A**–**D**) Panel shows the immunodetection of METTL7A using monoclonal antibody (green) and GFAP using polyclonal antibody (red). (**A**) METLL7A labeling distributed across the molecular layer (arrows) and strong immunoreactivity in the apical region (arrowheads). (**A′**) Immunodetection of GFAP in Bergmann glia (arrows) and higher immunodetection in the apical regions of molecular layer (arrowheads). (**A‴**) Colocalization of METTL7A and GFAP in the Bergmann glia and strong immunodetection in the apical region of this layer where the Bergmann glia constitutes the glia limitans (arrowheads). (**B**) Panel shows the immunoreactivity in the Purkinje layer of METTL7A, possibly in the Bergmann glia (arrowheads). (**B′**) Note positive cells for GFAP in the Purkinje layer (arrowheads). (**B‴**) Colocalization of METTL7A and GFAP in the Purkinje layer, possibly in the cytoplasm of Bergmann glia by its perinuclear immunoreactivity (arrowheads). (**C**) Low immunoreactivity in cells of the granular layer (arrowheads). (**C′**) Strong immunodetection of GFAP in the cells of the granular layer. (**C‴**) Merge of METTL7A and GFAP in the granular layer; arrowheads indicate colocalization of METTL7A and GFAP. (**D**) Panel shows the expression of METTL7A in white matter (arrowheads). (**D′**) Note cells positive for GFAP (arrowheads) in white matter. (**D‴**) Merge of METTL7A and GFAP in white matter shows colocalization of the two proteins (arrowheads) and expression of GFAP (arrows). (**A″**,**B″**,**C″**,**D″**) Nuclei stained with Hoechst 33342 (blue). (**A**–**D**) Tissue thickness—7 µm. Scale bar—4 µm, 20× magnification. Insets are zoomed in twofold. **CSF**: cerebrospinal fluid; **GL**: granular layer; **Glia L**: glia limitans; **PL**: Purkinje layer; **PM**: pia mater; **ML**: molecular layer; **WM**: white matter.

To further characterize the high immunoreactivity in the molecular layer, we carried out double staining for METTL7A and GFAP, performing the latter to label glial cells. We used polyclonal and monoclonal antibodies for METTL7A and GFAP, respectively (Figure 2), and also the converse combination (Figure 3). Our results showed that METTL7A colocalizes with GFAP in the molecular layer (0.72 ± 0.03 Pearson’s coefficient (PC), Figure S3A), where projections of Bergmann glial cells are found (Figure 2A–A‴,B–B″ and Figure 3A–A‴, and Movie S1). METTL7A also showed a low cytoplasmic signal in GFAP-positive cells at the edge of the Purkinje layer, corresponding to the cell body of Bergmann cells (Figure 2C–C‴ and Figure 3B–B‴) and strong immunoreactivity in the end-feet of Bergmann glial cells at the edge of the molecular layer, in intimate contact with the pia matter (Figure 2B–B‴ and Figure 3A–A‴). Both structures contribute to create a physical barrier that separates the cerebellar tissue from CSF. The granular layer (Figure 2D–D‴ and Figure 3C–C‴) shows the co-expression of METTL7A and GFAP in some astrocytes (0.59 ± 0.03 Figure S3A). Figure 2E–E‴ and Figure 3D–D‴ show the immunoreactivity of METTL7A and GFAP in white matter and their colocalization in some cells (0.51 ± 0.02 PC Figure S3A).

### 2.3. Co-Immunostaining of METTL7A and Tuj1 in Human Cerebellar Tissue 

**Figure 4:**
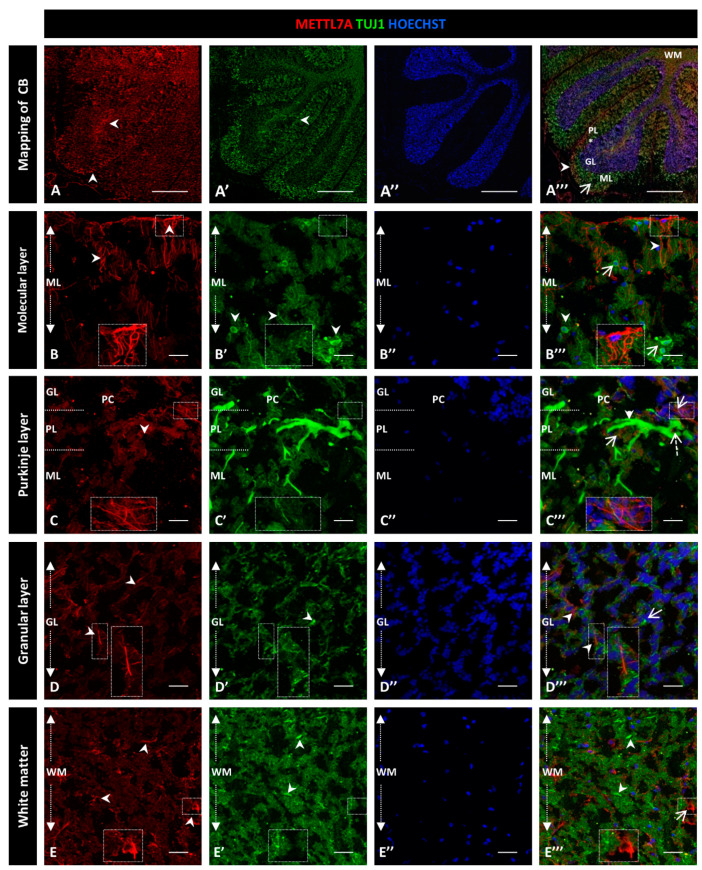
Immunodetection of METTL7A and Tuj1 in human cerebellum. (**A**–**E**). Image shows the immunodetection of METTL7A using polyclonal antibody (red) and Tuj1 using monoclonal antibody (green). (**A**–**A**‴) Mapping of adult human cerebellum. (**A**) Immunodetection of METTL7A (arrowheads). (**A′**) Immunoreactivity for Tuj1 (arrowheads). (**A**‴) Merge of METTL7A (arrowheads) and Tuj1 (arrow). Asterisk indicates PL. (**B**) Immunoreactivity of METTL7A distributed across the molecular layer and apical region (arrowheads). (**B′**) Immunodetection of Tuj1 in the molecular layer (arrowheads). (**B**‴) Image shows non-colocalization of METTL7A (arrowheads) and Tuj1 (arrows). (**C**) Low immunoreactivity of METTL7A in the Purkinje layer (arrowheads). (**C′**) Tuj1 positive cells in the Purkinje layer. (**C**‴) Positive immunodetection of some Purkinje cells for METTL7A in the cytoplasm (dotted arrow) and immunodetection of Tuj1 in Purkinje cells (arrowhead). Arrows indicate METTL7A immunodetection. (**D**) Low immunoreactivity of METTL7A in the granular layer (arrowheads). (**D′**) Immunodetection of Tuj1 in the granular layer (arrowheads). (**D**‴) Merge of METTL7A and Tuj1 in the granular layer; arrowheads indicate immunodetection of METTL7A and arrows indicate immunodetection of Tuj1. (**E**) Panel shows the expression of METTL7A in white matter (arrowheads). (**E′**) Immunodetection of Tuj1 in the white matter (arrowheads). (**E**‴) Merge of METTL7A and Tuj1 in the white matter; arrows indicate immunodetection of METTL7A and arrowheads indicate immunodetection of Tuj1. (**A″**,**B″**,**C″**,**D″**,**E″**) Nuclei were stained with Hoechst 33342 (blue). (**A**–**E**) Tissue thickness—7 µm. (**A**) Scale bar—1 mm, 10× magnification. (**B**–**E**) Scale ba—4 µm, 20× magnification. Insets are zoomed in twofold. **CB**: cerebellum; **GL**: granular layer; **ML**: molecular layer; **PL**: Purkinje layer; **WM**: white matter.

To explore whether METTL7A localizes in neurons, we performed a co-immunostaining with Tuj1 as a neuronal marker (Figure 4A–A‴). Our analysis showed subtly detectable colocalization of METTL7A and Tuj1 in the molecular layer (0.40 ± 0.04 PC Figure S3B; Figure 4B–B‴). However, our analysis showed that METTL7A is expressed at low levels in the soma and in projections of some Tuj1-positive cells in the Purkinje layer (0.58 ± 0.06 PC Figure S3B; Figure 4C–C‴ and Figure S4A–A‴). Thus, the major expression of METTL7A was in astrocytes, particularly in the glia limitans formed by Bergmann glial cells. The granular layer showed low co-reactivity for METTL7A and Tuj1 (0.45 ± 0.04 PC Figure S3B; Figure 4D–D‴), and white matter cells showed reactivity for METLL7A and Tuj1 but not co-expression (0.34 ± 0.05 PC Figure S3B; Figure 4E–E‴).

### 2.4. Three-Dimensional Reconstruction Analysis

**Figure 5:**
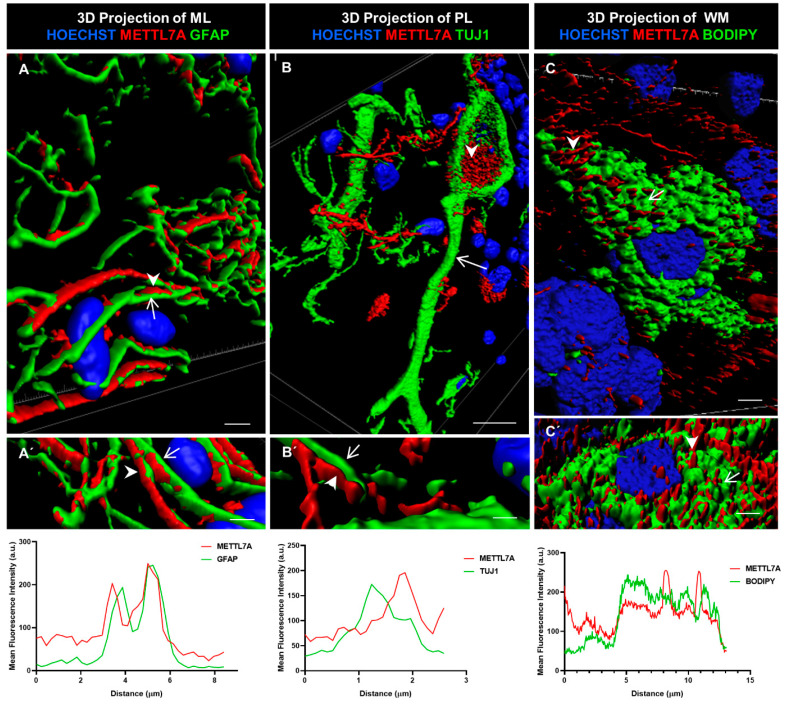
Three-dimensional rendering model and comparative profiling in longitudinal confocal axis. (**A**,**A**′) These images show the localization of METTL7A (red; arrowhead) and GFAP (green; arrow). Bottom graph shows mean fluorescence intensity (a.u.) for both proteins. Analysis of mean fluorescence intensity was performed following a line between the arrowhead and the arrow. (**B**) Purkinje cells (Tuj1-positive cell, green; arrow) show strong reactivity for METTL7A (red; arrowhead) in droplets in their cytoplasm. (**B**′) Image shows small contacts of Bergmann glia (arrowhead) with a neuronal projection (arrow). Bottom graph shows mean fluorescence intensity (a.u.) for both proteins. Analysis of mean fluorescence intensity was performed following a line between the arrowhead and the arrow. (**C**,**C**′) A white matter cell. The panel shows a lipid-droplet-filled cell which is marked with BODIPY (green; arrow) and METTL7A (red; arrowhead). Bottom graph shows mean fluorescence intensity (a.u.) for both proteins. Analysis of mean fluorescence intensity was performed following a line between the arrowhead and the arrow. Tissue thickness for (**A**–**C**) is 7 µm. (**A**) Scale bar—4 µm and (**A**′) 3 µm (high magnification 63×). (**B**) Scale bar—10 µm (high magnification 63×). (**B**′) Scale bar—2 µm (high magnification 63×). (**C**) Scale bar—5 µm (high magnification 100×). (**C**′) Scale bar—5 µm (high magnification 63×).

We further characterized METTL7A localization using 3D rendering. This method involves creating a solid mask for each channel used to demarcate the surface for each labeling, allowing us to visualize the volume of the labeled structure. In addition, we quantified the colocalization of METTL7A with each marker. We found that METTL7A was present along of the cellular projections of Bergmann cells (Figure 5A,A′), in multiple contacts between Bergmann glial cells and Purkinje cells (Figure 5B,B′), and in intracellular droplets in some Purkinje neurons (Figure 5B,B′ and Movie S2). A quantification analysis showed a high overlap between the GFAP and METTL7A intensity profiles (Figure 5A′, lower panel). However, a non-overlapping pattern was identified for the intensity profiles for METTL7A and TUJ1 (Figure 5B′, lower panel), indicating that another cell type positive for METTL7A makes contacts with TUJ1-positive neurons. After staining with BODIPY 493/503 to detect lipid droplets, METTL7A also appeared in cytoplasmic lipid droplets in some white matter cells (Figure 5C,C′ and Figure S4B–B‴, and Movie S3). The intensity profiles of BODIPY and METTL7A were partially overlapped (Figure 5C′, lower panel).

## 3. Discussion

In this study, we address for the first time METTL7A localization in the human cerebellum. METTL7A is a poorly studied member of the METTL family of methyltransferases whose expression profile in the brain and its function were completely unknown. Here, we have found METTL7A in the end-feet of GFAP-positive Bergmann glial cells, a structural component of the CSF–brain barrier, and also in their contacts with Purkinje neurons. METTL7A was also detected in one of its subcellular localizations, the lipid droplets of some cells in the white matter. These results could suggest a possible role of METTL7A in the CSF–brain barrier and also in the modulation of the synaptic activity of Purkinje neurons.

METTL7A could have a structural function in astrocytes rather than the typical neutrophil degranulation role assigned by Gene Ontology tools. In our study, METTL7A colocalized with the glial marker GFAP, showing high expression in Bergmann glial cells and the glia limitans superficialis in the cerebellum. In addition, our search in open online databases with single-cell transcriptomic data also showed a high expression of METTL7A in Bergmann glia and to a lower extent in astrocytes. These findings are consistent with a previous transcriptome study, which showed that METTL7A is highly expressed in astrocytes [28]. Bergmann glia carry out several functions during cerebellar development. For example, they are essential for the correct migration of granular cells [29] and participate in dendrite formation in Purkinje cells [30,31]. These cells are also intimately related to the pia mater in the cerebellum to build the inner CSF–brain barrier. The pia mater is separated from the cerebellar parenchyma by a physical and immunological barrier formed by the end-feet of the Bergmann glial cells where METTL7A is highly expressed. Th end-feet of the Bergmann glial cells separate the cerebellar parenchyma into two immune compartments: the immune-privileged parenchyma in which the glia limitans release anti-inflammatory factors and the non-immune privileged subpial space towards which these cells secrete pro-inflammatory molecules [32]. The integrity of this barrier is essential for the correct functioning and homeostasis of the cerebellum [33]. In this regard, the subcellular localization of other proteins such as aquaporin-4 is involved in the regulation of the BBB’s integrity [34,35,36,37,38,39]. Thus, METTL7A could have a role in the protection of the brain parenchyma via Bergmann glia. However, further studies are needed to better characterize the possible specific effect of METTL7A on the integrity of the CSF–brain barrier.

Although no previous studies are available on the function of METTL7A in the brain, other members of the METTL family have been reported to be involved in the correct proliferation of cerebellar granule cell progenitors, Purkinje cell maturation, and Bergmann glia organization pattern during cerebellar development [40]. Other studies reported that Purkinje neurons could mediate Bergmann glia cell development and maturation through Notch [41] and Sonic hedgehog signaling [42]. In this study, we identified that METTL7A is in the contact between Bergmann glial cells and Purkinje neurons. Grosche and colleagues [43] proposed that Bergmann glia have microdomains that could confine the neurotransmitter release to a specific area during the glia–neuron interaction and increase the synaptic efficiency. In this regard, they proposed that Bergmann glia microdomains could recognize independent synaptic spines of Purkinje neurons. The function of Bergmann glia microdomains could be the recognition of a specific spine type of Purkinje neurons to increase the synaptic efficiency between these two types of cells in the cerebellum. Purkinje neurons integrate synaptic inputs from many granule neurons to send an output signal to cortical regions. Thus, Purkinje neurons are one of the key components of internal cerebellar circuits. In addition to this, we detected a low expression of METTL7A in TUJ1-positive Purkinje neurons, which was backed up by a previous single-cell analysis in the mouse brain (Supplementary Data S1) [27]. Our identification of a novel METTL7A localization in the contacts between the Bergmann glia and the Purkinje neurons could be important for the synaptic activity of Purkinje neurons, with an impact on the activity of internal cerebellar circuits. However, further studies are required to explore this possibility.

METTL7A has been described as modulated by androgens in human neural stem cells, particularly by testosterone [44]. Subtle hormonal imbalances during periods of the intrauterine life could alter normal neuronal development. This contributes to an increase in the susceptibility of male individuals to develop neurodevelopmental disorders such as autism and psychotic spectrum disorders. Indeed, METTL7A expression levels have previously been investigated in brain tissue in schizophrenia. A recent proteomic study found a reduction in METTL7A protein levels in the cerebellar cortex in schizophrenia patients and in the schizophrenia murine models [14]. In our study, we found that the main source of METTL7A in the cerebellar cortex is in the GFAP-positive cells. Thus, the reduction in METTL7A protein reported in the cerebellum in schizophrenia is more than likely due to a reduction in this protein in astrocytes and more extensively in Bergmann glial cells. Other studies have reported a decrease in METTL7A RNA and protein levels on the prefrontal cortex (Brodmann area 46/10) and the anterior cingulate cortex (Brodmann area 24) in schizophrenia subjects, respectively [15,45]. Another study on the prefrontal cortex (Brodmann area 9) reported an increase in RNA levels [16]. In addition, the loss of astroglial domains related to cognitive dysfunction in schizophrenia has been described [46]. Our study found that METTL7A is localized in both the astroglial microdomains with Purkinje cells and in the end-feet of Bergmann glia, which are a key element in the CSF–brain barrier to avoid the entrance of inflammatory molecules into the brain. The altered integrity of the brain barrier has been described in several brain regions in schizophrenia [47,48]. A pro-inflammatory brain state has been linked to schizophrenia, including in the cerebellum [21], with a possible role of the glia limitans in this process. Although other studies are needed in this context, the downregulation of METTL7A linked to stress in schizophrenia could have a possible impact on the integrity of the CSF–brain barrier and in the synaptic activity of Purkinje cells, the sole output of the internal cerebellar circuits to the cortex. 

In our study, we also observed that METTL7A localizes in lipid droplets in white matter cells. This result is in line with the role of METTL7A in lipid droplet formation [5]. However, the function and dynamics of METTL7A in lipid droplets are still not totally well understood. Further studies will be needed to explore the possible regulation of METTL7A in the context of lipid droplet biogenesis. Lipid-droplet-containing microglia have been described during aging and have been linked to inflammation and cognitive decline [49], both processes which are also related to schizophrenia [18,19,20,21,50]. A previous study on METTL7A protein levels in the cerebellum in schizophrenia only included grey matter, while in stress animal models, the whole cerebellum was used [14]. A smaller decrease in METTL7A was found in the whole cerebellum than in the grey matter, raising the possibility that a different deregulation of METTL7A could occur in white matter. Further investigations in white matter will help to elucidate the possible deregulation of METTL7A in this cerebellar region in schizophrenia.

In summary, we reveal a novel localization of METTL7A in the cerebellar human glia limitans. In this study, we found a high expression of METTL7A in the end-feet of Bergmann glia and in the contacts of these cells with Purkinje neurons, suggesting that METTL7A could participate in the maintenance of the cerebellar parenchyma homeostasis and in the modulation of Purkinje neuronal activity.

## 4. Materials and Methods

### 4.1. Postmortem Human Brain Tissue 

Postmortem adult human brain tissue from the cerebellum of a healthy 47-year-old male subject (PMD = 4.92 h; pH = 6) was obtained from the neurologic tissue collection of the Institute of Neuropathology of the Hospital Universitari de Bellvitge. The brain was obtained following donation according to the Spanish legislation and the guidelines and protocols of tissue donation for research approved by the local ethics committee of the Hospital Universitari de Bellvitge. The brain sample selection was based on: (1) an age lower than 50 years, (2) no history of mental disorder, neurological disorder, or drug abuse, (3) no accidental or natural cause of death that compromised the integrity of the brain region of interest, (4) the absence of tumor or hemorrhage in the brain region of interest, and (5) a brain pH higher than 5.5. The study was approved by the Institutional Ethics Committee of Parc Sanitari Sant Joan de Déu. Specimens extending from the pial surface to white matter were dissected and stored at −80 °C.

### 4.2. Immunohistochemistry of Human Cerebellar Tissue 

Frozen tissue was embedded in an optimal cutting temperature compound (OCT) and cut in cryostat (Microm HM 525, Thermo Scientific, Waltham, MA, USA). The frozen block was oriented to obtain 7 μm thick sagittal sections. The sections were fixed in 4% paraformaldehyde for 30 min at room temperature (RT) and washed in phosphate-buffered saline (PBS pH 7.4 (Sigma, St. Louis, MO, USA)). They were then incubated with blocking solution (PBS containing 15% goat serum, 0.05% BSA, and 0.05% Triton X-100) for 1 h at RT. The sections were immunostained with rabbit anti-METTL7A (A8201, dilution 1:75; ABclonal, Woburn, MA, USA), mouse anti-METTL7A (AB128017 (Clone 87.1_1E7), ABclonal, Woburn, MA, USA), dilution 1:75), mouse anti-Tuj1 (MMS435P, dilution 1:500; Covance, San Diego, CA, USA), and mouse anti-GFAP (MAB360, dilution 1:400; Millipore, Burlington, MA, USA). These antibodies were diluted in PBS containing 1% goat serum, 0.05% BSA, and 0.05% Triton X-100. For the double immunostaining of METTL7A and GFAP, we first incubated the samples with anti-METTL7A overnight at 4 °C and then with anti-GFAP for 45 min at 4 °C. As secondary antibodies, we used goat anti-rabbit Cy3 (Dilution 1:500, Amersham Biosciences, Piscataway, NJ, USA) and the goat anti-mouse Dye Light 488 (35503, dilution 1:500; Invitrogen, Waltham, MA, USA). The nuclei were visualized with Hoechst 33342 (Invitrogen, dilution 1:400). For lipid droplets, we immunostained without permeabilization, first with anti-METTL7A for 48 h at 4 °C and then with the secondary antibody for 1 h, followed by a final incubation for 30 min at RT with 20 µg/mL of BODIPY 493/503 (Thermofisher Scientific, Molecular Probes) and Hoechst 33342. The sections were mounted with a prolong diamond antifade mountant (Invitrogen P36970).

### 4.3. Confocal Microscopy

A confocal microscopy analysis was performed using a Leica TCS SP8 STED 3X equipped with a white light laser confocal microscope, HyVolution mode, and hybrid detectors (Leica Microsystems GmbH, Mannheim, Germany). For extended-volume imaging at a low magnification (HC PL APO CS2 10×/0.4 dry), a high-precision motorized stage was used to collect the large-scale 3D mosaics of each tissue section. The software automatically generates a list of 3D stage positions covering the volume of interest, which are computed using the dimensions of a single image in microns and the degree of overlap between adjacent images. Individual image tiles were 696 × 696 pixels, and the z-step was 2.5 μm. A total of 49 stacks were captured for each extended image. The brain tissues were excited sequentially at three different wavelengths: 405 nm, 488 nm, and 552 nm, which respectively excite Hoechst 33342, GFAP/TUJ1 and BODIPY, and METTL7A. Hoechst 33342 was detected in the 420–465 nm range, GFAP/TUJ1 and BODIPY in the 500–550 nm, and METTL7A in the 565–700 nm. Z-stacks were acquired at 20×, 63×, or 100× magnification, with 0.4 and 1.4 numerical aperture objective lenses, respectively. At a low magnification, 10 sections were acquired every 1 μm across the tissue thickness. For volume rendering in higher magnification stacks, it was necessary to optimize z-sectioning, and the z-step size was set to 0.3 μm. The images were then deconvolved using Huygens software (SVI, Leiden, The Netherlands). The 3D models were generated using Imaris x64 v7.2.1 software (Bitplane, Zurich, Switzerland) with Surpass Mode. Maximum projections were created using LAS AF™ software (Leica Microsystems, Heidelberg, Germany). For each figure and in order to compare the confocal data, identical confocal settings were used for the image acquisition of different experiments.

### 4.4. Quantitative Colocalization

To quantify the degree of colocalization between the different layers of the cerebellum (molecular and granular layers and the white matter), the Pearson coefficient (PC) was determined from the corresponding confocal images using FIJI software (ImageJ, National Institutes of Health, Bethesda, MD, USA) with JACoP plugin (Just Another Colocalization Plug-in) [51]. A colocalization analysis in the Purkinje layer could not be performed because of the sparse distribution of Purkinje neurons, the small area of the layer, and the close proximity of astrocytes and neurons from other layers. Fluorescence intensity profiles of the linear region of interest (ROI) from METTL7A-GFAP, METTL7A-TUJ1 and METTL7A-Bodipy were also evaluated. Data were analyzed and visualized using LAS X software, version 3.5.7 (Leica Microsystems, Wetzlar, Germany), and GraphPad Prism 6 software (version 6, GraphPad Software, New York City, NY, USA). A nonparametric paired Friedman test was performed, where appropriate.

## Data Availability

The data that support the findings of this study are available from the corresponding author upon reasonable request.

## Supplementary Materials

The supporting information can be downloaded at: https://www.mdpi.com/article/10.3390/ijms24098405/s1.
