# Supplementary material for: A Novel Localization of METTL7A in Bergmann Glial Cells in Human Cerebellum
Source: Int J Mol Sci. 2023 May 7;24(9):8405. doi: 10.3390/ijms24098405 (PMC10179429; doi:10.3390/ijms24098405)
Supplement: Supplementary file 1 [file ijms-24-08405-s001.zip › Vera et al_Supplementary Material_Rev_Accepted changes.pdf]

Supplementary Material and Methods

Bioinformatic analysis

To analyze the specific cell expression pattern of cerebellar METTL7A in open online resources, we used the Human Cell Landscape (Han et al., 2020) and DropViz (Saunders et al., 2018) softwares. The former is an online open software about single cell expression profiles of human cells from distinct organs. This database is build by 700000 single cells from more than 50 human tissues and cell cultures. The latter is an online open software about single cell expression profiles in the mouse brain. This database is build by 690000 individual cells from nine different regions of the adult mouse brain, identifying 1.45 billion RNA transcripts. The search of METTL7A gene was done in both databases. For the Human Cell Landscape software, Brain-Adult-Cerebellum was selected for this search.

Supplementary Figures

Figure S1

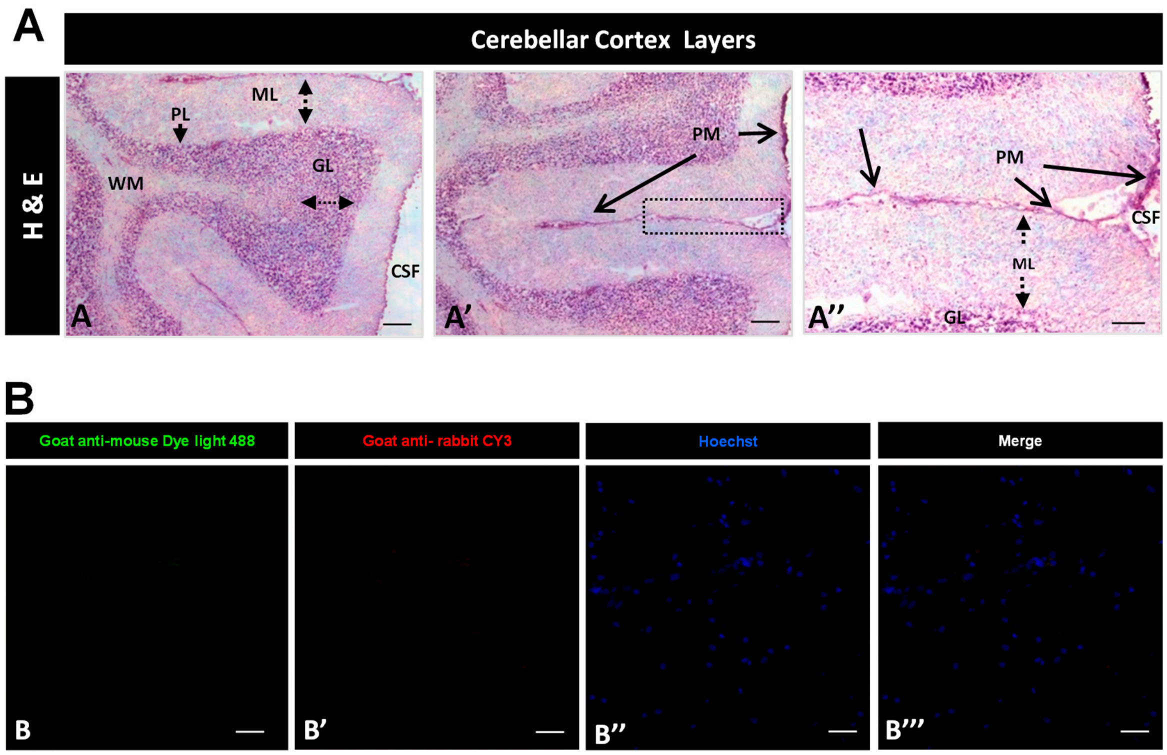

Figure S1. Haematoxylin-Eosine staining and negative control for primary antibodies. **A-A'**. Image shows Haematoxylin-Eosine stain of human cerebellum showing the good preservation of the tissue and its layers. Image **A''** shows the 20x magnification of the square labelled in **A'**. **GL**: Granular layer; **PL**: Purkinje layer; **ML**: Molecular layer; **WM**: White matter; **PM**: Pia mater; **CSF**: Cerebrospinal fluid. **A-A'**: Tissue thickness 7 μm. Scale bar 5 μm and 10x magnification. **A''**. Tissue thickness 7 μm. Scale bar 5 μm and 20x magnification. **B-B'**. Negative control for secondary antibody goat anti-mouse Dye light 488 and goat anti-rabbit Cy3 (**B'**) as negative control for rabbit anti-METTL7A, mouse anti-GFAP and mouse anti-Tuj1. Negative control of Figure 5, S4-S9. All negative controls were used without incubating the tissue with the corresponding primary antibody. Nuclei stained with Hoechst (**B''**). The images

were merged (**B'''**). **B-B'''**. Tissue thickness 7 µm. Scale bar 4 µm. Images were acquired at 20x magnification.

Figure S2

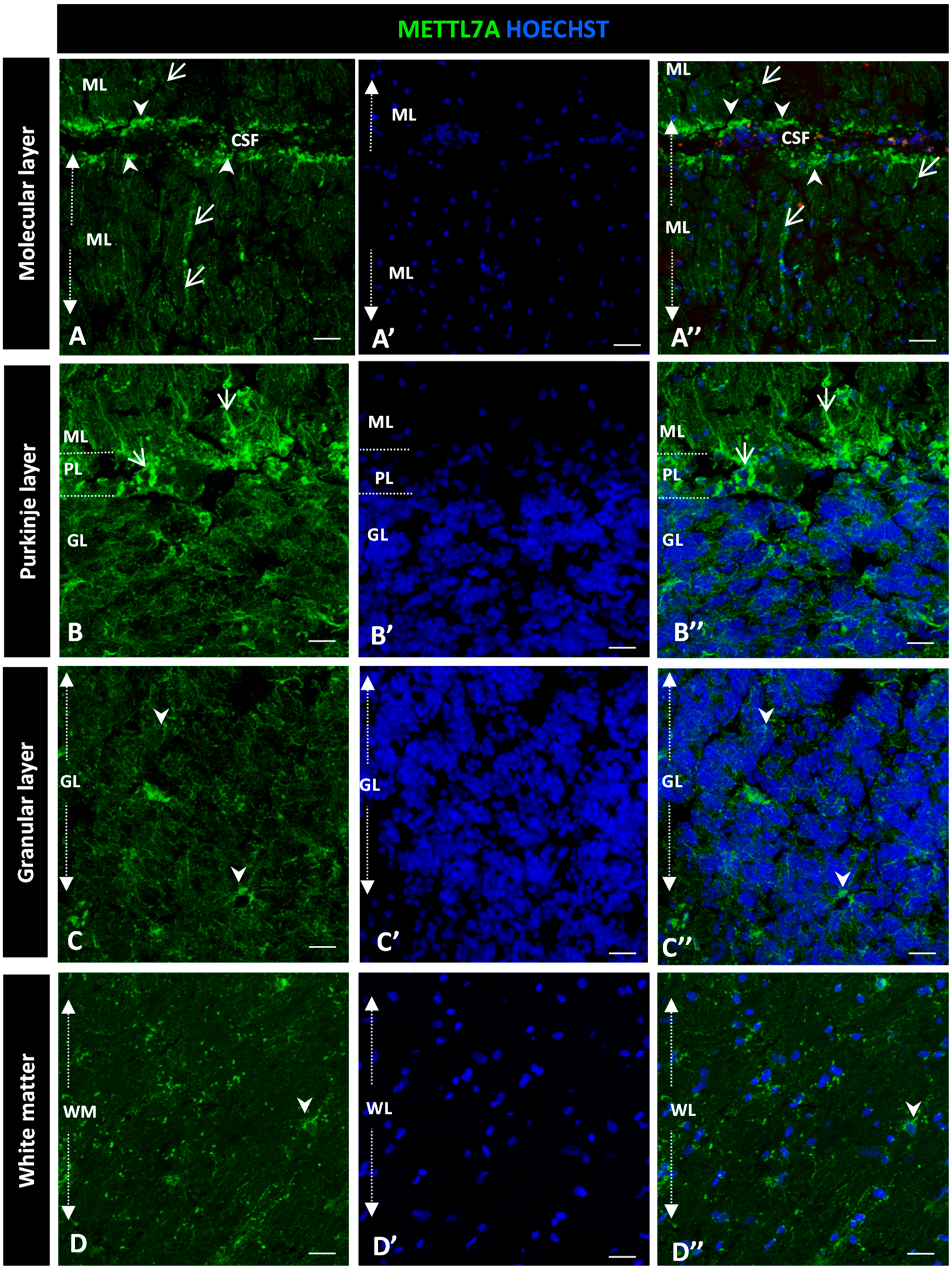

Figure S2. Immunohistochemistry of METTL7A in the human cerebellum. **A-D**. Panels show the immunodetection of METTL7A using monoclonal antibody AB128017 (Clone 87.1_1E7) with a similar expression profile of METTL7A as in Fig. 5. **A-A''**. Immunodetection of METTL7A in the molecular layer (arrows) and high immunoreactivity in the apical region (arrowheads). **B-B''**. Purkinje layer projection shows reactivity of METTL7A possibly in the Bergmann glia cells (arrows). **C-C''**. Immunoreactivity of METTL7A in the cells of the granular layer (arrows). **D-D''**. Image of white matter showing cells positive for METTL7A (arrowheads). **A'**, **B'**, **C'** and **D'**. Nuclei were stained with Hoechst. **GL**: Granular layer; **PL**: Purkinje layer; **ML**: Molecular layer; **WM**: White matter; **PM**: Pia mater; **CSF**: Cerebrospinal fluid. Tissue thickness 7 μm. Scale bar 4 μm, 20x magnification.

Figure S3

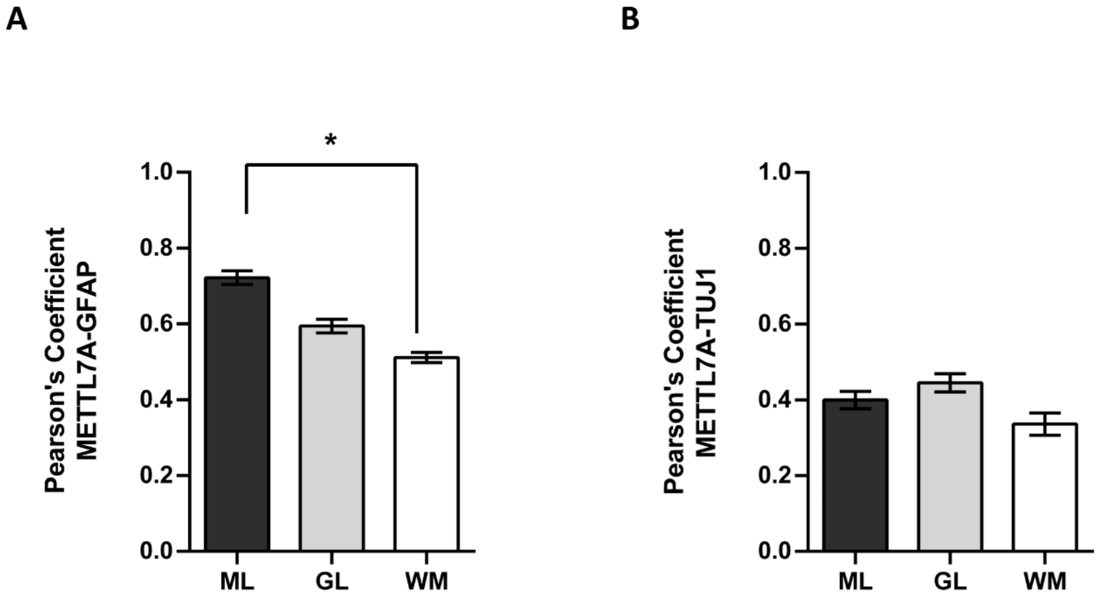

Figure S3. Colocalization analysis of METTL7A with neural markers in the distinct cerebellar layers. **A**. Pearson's coefficient of METTL7A colocalization with the astroglial marker GFAP. **B**. Pearson's coefficient of METTL7A colocalization with the neuronal marker TUJ1. Nonparametric paired Friedman analysis was performed; *p*-value *<0.05. **GL**: Granular layer; **ML**: Molecular layer; **WM:** White matter.

Figure S4

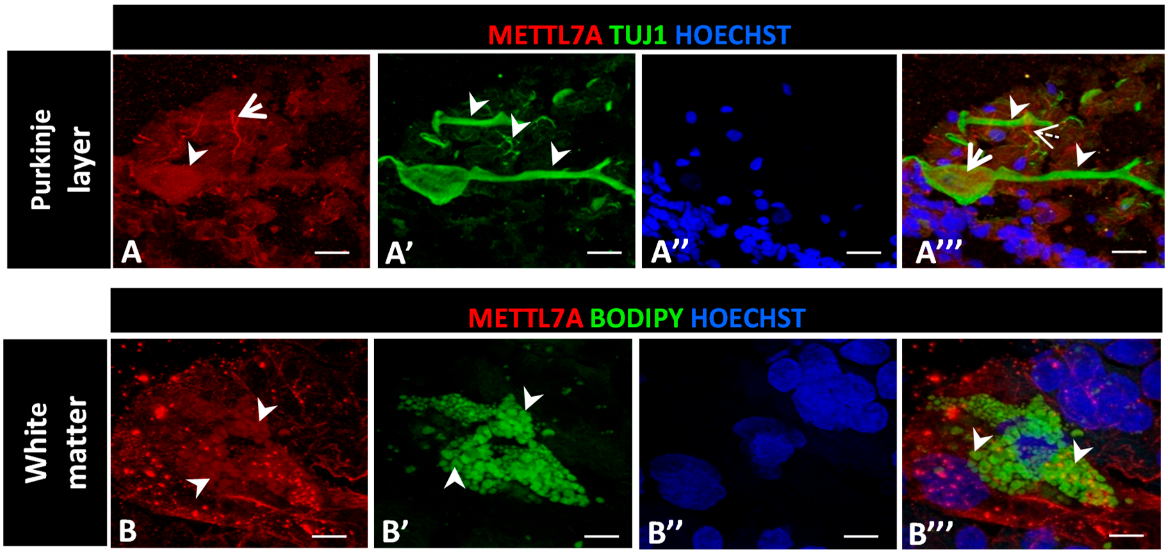

Figure S4. Immunohistochemistry of METTL7A, Tuj1, and BODIPY stain. **A-A’’’**. Images show immunodetection of METTL7A and Tuj1 in a Purkinje cell. **A**. Low immunoreactivity of METTL7A in a Purkinje cell (arrowheads) and fibres in the Purkinje layer (arrow). **A’**. Immunodetection of Tuj1 in a Purkinje cell (arrowheads). **A’’’**. Low immunoreactivity of METTL7A in the cytoplasm of a Purkinje cell (arrow), fibre positive for METTL7A (dotted arrow) in the Purkinje layer and immunoreactivity of Tuj1 in a Purkinje cell (arrowheads). **B-B’’’**. Images show BODIPY 493/503 staining with immunodetection of METTL7A in white matter. **B**. Image shows immunodetection of METTL7A in lipid droplets (arrowheads) contained in the cells of white matter. **B’**. BODIPY stain for lipid droplets (arrowheads) in a cell of white matter. **B’’’**. Merge of METTL7A and BODIPY and their colocalization in lipid droplets. All images of the different tissue layers were acquired using the same conditions (laser intensity and exposition time). **A’’** and **B’’**. Nuclei were stained with Hoechst. Tissue thickness 7 µm. Scale bar 4 µm. **A-A’’’**. 63x magnification. **B-B’’’**. 100x magnification.

Movie S1. Immunodetection of METTL7A (red) and GFAP (green) in a 3D rendering model of the human cerebellar molecular layer. Nuclei (blue) are stained using Hoechst 33342.

Movie S2. Immunodetection of METTL7A (red) and Tuj1 (green) in a 3D rendering model of the human cerebellar Purkinje neuronal layer. Nuclei (blue) are stained using Hoechst 33342.

Movie S3. Immunodetection of METTL7A (red) and BODIPY (green) in a 3D rendering model of the human cerebellar white matter. Nuclei (blue) are stained using Hoechst 33342.
